# Blended learning during and beyond the COVID-19 pandemic: Attitudes of nurse educators in Gauteng

**DOI:** 10.4102/hsag.v28i0.2194

**Published:** 2023-06-02

**Authors:** Sarah Namulondo, Melitah M. Rasweswe, Ramadimeja S. Mooa

**Affiliations:** 1 Department of Nursing Science, Faculty of Health Sciences, University of Pretoria, Pretoria, South Africa; 2 Department of Nursing Science, Faculty of Health Sciences, University of Limpopo, Polokwane, South Africa

**Keywords:** attitudes, blended learning (BL), COVID-19, nurse educator, Gauteng

## Abstract

**Background:**

The use of blended learning (BL) pedagogy has become inevitable due to contemporary technological innovations in the nursing education sector. As of late, the need to use BL pedagogy has resulted by the sudden occurrence of the COVID-19 pandemic. However, several nurse educators still experience uncertainties in using BL due to technological, psychological, infrastructure and equipment readiness barriers.

**Aim:**

To report the attitudes of nurse educators towards the use of BL pedagogy as a new norm of teaching and learning in public nursing education institutions (NEIs) in the Gauteng Province (GP), South Africa, during and beyond the COVID-19 pandemic period.

**Setting:**

The study was conducted in five Gauteng public NEIs.

**Methods:**

A descriptive non-experimental quantitative design was conducted with 144 nurse educators. Data was collected through a questionnaire. Statistical Analysis Software (SAS) was used to analyse data with the help of a biostatistician.

**Results:**

Technologically, only 50% (*N* = 72) found BL easy to use while 48% (*n* = 69) were ready and willing to use the BL Psychologically, more than half, that is, 65% (*n* = 94) lacked the confidence to use BL pedagogy. About 55% (*n* = 79) reported having inadequate BL infrastructure, while 32% (*n* = 46) seemed to be satisfied with the availability of effective equipment to support BL pedagogy.

**Conclusion:**

Based on the results, it is apparent that nurse educators in Gauteng are not technologically and psychologically ready, since the infrastructure and equipment to support the BL are not adequately provided.

**Contribution:**

The study emphasised the purpose of performing regular assessments to establish the overall readiness of nurse educators to successfully implement the BL pedagogy.

## Introduction

Blended learning (BL) has improved the performance of many educational activities and influenced learning institutions to develop new teaching and learning methods in the fourth industrial revolution (4IR) (Zainuddin & Muftia 2018). This educational pedagogy involves the integration of both traditional face-to-face learning and e-learning to complement the benefits of both designs (Ndayisenga et al. 2021). In addition, through BL, the course materials may be presented online and from multi-sources (Zainuddin & Muftia 2018). Karma, Darma and Santiana (2021) are of the opinion that integrating BL in education is necessary to cater for the technology generation and can help to bring quality education to all, including those who were born before technology. Furthermore, it was also found that BL provides educators with tools to make learning interactive and interesting for both educators and students as both parties become actively involved in integrating teaching and learning (Zainuddin & Muftia 2018). In addition, Ndayisenga et al. (2021) affirmed that if BL is implemented appropriately, it has the potential to stimulate the development of higher cognitive skills and the ability to deliver deeper learning attitudes and acquisition skills that are beneficial for application in the clinical setting.

Therefore, it is apparent that this teaching and learning method is a necessity for nursing education. If implemented well, it will support nursing students to learn independently, as well as with fellow students, especially during experiential learning. Hence, the use of BL pedagogy in nursing education has internationally gained popularity due to several contemporary trends in the nursing profession, thus, making it a mandatory norm for all nurse educators to be ‘digitally elite’. Lately, the use of BL has further been precipitated by the lockdown restrictions caused by the emergence of the coronavirus disease 2019 (COVID-19) pandemic, which changed many ways in which teaching and learning in nursing education institutions (NEIs) is conducted (Jowsey et al. 2020; Ndayisenga et al. 2021). Since the COVID-19 pandemic, most of the NEIs have resorted to the adoption of BL, to sustain the continuity of their teaching activities both during and beyond the COVID-19 pandemic (Mishra, Gupta & Shree 2020). Suleiman, Yahya and Tukur (2020) stressed that BL assisted in breaking the barriers caused by the lockdown restrictions.

The current nurse educators are therefore required to be competent in using BL tools during classroom interactions to make learning more meaningful (Agu et al. 2021). They are expected to be able to restructure teaching strategies, skills and creativity to enhance transition readiness towards the use of BL (Agu et al. 2021). Despite the reported benefits of BL in education and learning, as well as the requirements and expectations to know and use BL pedagogy in nursing education, there are still nurse educators who are not implementing it (Mncube, Olawale & Hendricks 2019). The reason might be that the nurse educators are not well prepared and ready. This is of much concern, especially in Africa which is still in the technological transition stage (Baloyi, Jarvis & Mtshali 2022).

Globally, prior studies reported several attitudes of nurse educators towards the use of BL. A policy brief report about the delivery of education during the COVID-19 pandemic confirmed that educators across the world are not ready to effectively transition into using BL pedagogy. For example, a study conducted at a Malaysian university by Ooi, Balan and Saeed (2020) revealed that although the use of BL virtual platforms is a great invention, several educators expressed concerns about time intensiveness. There were additional challenges of inadequate technical support and difficulties in designing virtual learning experiences. A study conducted at Makerere University Medical School in Uganda showed that the institution was uncertain about implementing BL in teaching and learning due to insufficient infrastructure, equipment, information and communication technology (ICT) skills and psychological aspects (Olum et al. 2020).

In South Africa, during the peak of the COVID-19 pandemic in 2020, the NEIs were forced to adjust the methods of teaching and learning to online and BL, which can still be applicable even beyond the pandemic (Khan et al. 2022). However, several challenges such as negative attitudes emerged as barriers for the adoption of the BL pedagogy (Baloyi et al. 2022). Prior studies have also reported non-readiness of nurse educators in terms of psychological and technological capabilities, as well as equipment and environmental factors (Louw & Thukane 2019; Mncube et al. 2019). Because the use of BL pedagogy has become a mandatory norm during and beyond the COVID-19 pandemic, there is a need for a thorough assessment of nurse educators’ attitudes in terms of psychological, technical, equipment and environmental components, to improve the successful use of BL pedagogy in NEIs (Coopasami, Knight & Pete 2017). This paper reports the attitudes of nurse educators towards the use of the BL pedagogy, as a new norm of teaching and learning in public NEIs in Gauteng Province (GP), South Africa, during and beyond the pandemic period. The reported results are part of the larger study that was conducted as a requirement for the completion of a master’s degree.

## Aim of the study

This study aimed to report the attitudes of nurse educators towards the use of BL pedagogy as a new norm of teaching and learning in public NEIs in GP, South Africa, during and beyond the COVID-19 pandemic period.

## Material and methods

### Design

This study followed a quantitative descriptive non-experimental approach.

### Study setting

The study was conducted in five public NEIs, in GP, South Africa. These public NEIs offer a variety of undergraduate or post-basic courses in nursing qualifications, which are presented on a full-time basis.

### Study population and sampling

The study population were nurse educators (*n* = 500) from the five public NEIs in the GP, South Africa. The nurse educators who participated were actively teaching nursing students at all year levels of learning. The study sample was recognised using a two-stage sampling method, where the first stage applied the stratified sampling method that selected five public NEIs called the ‘stratums’. From within the ‘stratum’, a simple random sampling was then applied to select a representative sample from each NEI. Dividing out the population by strata aided researchers to easily choose the appropriate number of individuals from each stratum based on the proportions of the population. Furthermore, a simple random sampling without replacement method (SRSWOR) was used as the last stage of sampling to select nurse educators in each stratum. The SRSWOR method ensured that each nurse educator in the population had a known and equal probability to be selected and be part of the study. The SRSWOR method was done for each ‘stratum’. The process assisted in selecting the appropriate number of respondents from each ‘stratum’ based on the proportions of the population as indicated in Table 1. Based on the certain parameters (see notes below Table 1), for example, a sample size of 217 was determined (see Table 1).

**TABLE 1:** Determination of sample size.

Variable	NEI 1	NEI 2	NEI 3	NEI 4	NEI 5	Total
*N*_i_	109	93	98	100	100	500
*n*_i_	47	40	43	43	43	217

*Source*: Namulondo, S., Rasweswe, M M. & Mooa, R S., 2020, *Nurse Educators’ readiness to use blended learning in public nursing education institutions in Gauteng Province, South Africa*, viewed n.d., from https://repository.up.ac.za/handle/2263/78869

NEI, nursing education institution.

*n_i_* = sample size for each ‘stratum’; *n* = required sample size; *N_i_* = population size for each ‘stratum’.

### Instrument

A structured self-administered questionnaire was adopted from Trayek et al. (2016) and customised to suit the study. The biostatistician assisted in modifying some of the items. The questionnaire was divided into two sections, namely, section A and section B. Section A documented demographic data, which consisted of four questions: (1) gender, (2) age, (3) educational level and (4) employment classification. Section B comprised of an assortment of questions adopted from Chapnick’s readiness model (2000). The questions were divided into four readiness dimensions. Technological consisted of 12 items, psychological had 11 items, infrastructure had 7 items, and equipment preparedness consisted of 5 items. The 5-point Likert scale was used to measure all the items on nurse educator’s readiness to use BL. The questions were in English because it is the official language used in the public NEIs. The study supervisors and the biostatistician approved the questionnaire contents. Prior to the main study, the questionnaire was pretested among 15 nurse educators who did not form part of the final study. The pre-test revealed that a few questions were either ambiguous or partial, with a few duplications. The researchers refined new questions and included more relevant questions. The final questionnaire was more accurate, brief and clear, and consisted of different types of close-ended questions. The Cronbach alpha coefficient was used to determine the internal consistency of the questionnaire. The tool’s internal consistency yielded 0.83 or 83.16%.

### Data collection

Data were collected between June and August 2020. A questionnaire was used to collect data, either online (electronic) or paper-based (hardcopy). The electronic questionnaires were distributed and completed through the Qualtrics survey software system generated by the university. The researcher sent a survey link with the soft copy of the questionnaire, consent form and relevant instructions via email to the relevant institutional research committee chairpersons, who eventually distributed the link to the relevant respondents to complete. The survey link was specifically sent to the two NEIs, where physical access to the premises was not permitted due to COVID-19 restrictions. Upon completion, the results were secured in an electronic folder of a computer that can be accessed by the researcher only. The online data collection took approximately nine weeks to be finalised. The paper-based or hardcopy questionnaires were delivered to the NEI’s research chairperson by the first author per appointment, for the nurse educators to complete at their own convenient time. The NEI’s research chairperson distributed questionnaires to all the respondents who signed the informed consent form prior to completion. The completion took approximately 10–15 minutes. The completed questionnaires and signed consent forms were deposited in safely sealed boxes which were left in the NEIs for a period of three weeks. The first author collected the boxes on the agreed date.

### Data analysis

Qualtrics survey software system cleaned and analysed data completion and was extracted to an Excel sheet. Paper-based data were checked for completeness and captured using Microsoft Excel and converted into a *STATA 13* format for analysis by the biostatistician. Both analysed data from the Qualtrics survey software system and paper-based were captured on a Microsoft Excel spreadsheet and were used to determine the attitudes of nurse educators towards the use of BL. Descriptive statistics were used to combine, interpret and communicate numerical data. The descriptive statistics were presented in tables and graphs as frequencies and percentages.

## Results

### Demographical data

A total of 144 (*n* = 144) nurse educators participated in the study. There were 11% (*n* = 16) males, 88% (*n* = 126) females, while 1% (*n* = 2) did not respond to this question. The results indicated that most participants, that is, 32% (*n* = 46) were between the age group of 36–45 years, followed by 30% (*n* = 44) participants who were between 46–55 years of age. The participants who were above 55 years of age were 17% (*n* = 24) followed by those who are less than 35 years, 21% (*n* = 30). Most of the respondents, that is, 69% (*n* = 98) were lecturers. The majority of nurse educators, namely, 45% (*n* = 62) possessed a bachelor’s degree in nursing. The demographical data is presented in Table 2.

**TABLE 2:** Demographical data of the respondents (*N* = 144).

Variable	Frequency	Percentage
**Gender**		
Male	16	11
Female	126	88
Not responded	2	1
**Total**	**144**	**100**
**Age**		
≤ 35	30	21
36–45	46	32
46–55	44	30
> 55	24	17
**Total**	**144**	**100**
**Employment classification**		
Clinical facilitators	26	18
Head of Department	7	4
Lecturer	98	69
Nurse administrator	3	2
Preceptor	10	7
**Total**	**144**	**100**
**Educational level**		
Diploma in general nursing	3	1
Post diploma in public health	10	6
Clinical training in nursing	15	10
Bachelor’s degree in nursing	62	45
Master’s degree in nursing	46	34
PhD in nursing	8	4
**Total**	**144**	**100**

PhD, Doctor of Philosophy.

### Attitudes of nurse educators on blended learning

Respondents’ attitudes towards BL were investigated under technological, psychological, infrastructure and equipment.

#### Technological attitudes

The nurse educators were asked whether it is easy to use BL pedagogy or not. Half of the respondents, that is, 50% (*n* = 72) indicated that BL is easy to use, while 11% (*n* = 16) were uncertain and 39% (*n* = 56) did not find it easy to use. It was of significance to note that 48% (*n* = 69) of the respondents were ready and willing to use technical platforms for BL, 19% (*n* = 27) were uncertain, while 33% (*n* = 48) were not ready or willing to use BL. Refer to Table 3 for frequencies and percentages.

**TABLE 3:** Technological and psychological attitudes towards blended learning.

Technological attitudes of nurses on BL	Frequency (*N*)	%
**It is easy to use BL**		
Strongly agree	35	25
Agree	36	25
Uncertain	16	11
Strongly disagree	15	11
Disagree	39	28
No response	3	0
**Total**	**144**	**100**
**Readiness and willingness to use technical platforms for BL**		
Strongly agree	38	26
Agree	32	22
Uncertain	27	19
Strongly disagree	28	20
Disagree	19	13
**Total**	**144**	**100**
**Psychological attitudes towards BL**		
**Psychologically ready and willing to use BL**		
Strongly agree	29	21
Agree	41	28
Uncertain	24	17
Strongly disagree	27	18
Disagree	22	16
No response	1	0
**Total**	**144**	**100**
**Motivated to use BL**		
Strongly agree	21	15
Agree	40	27
Uncertain	29	20
Strongly disagree	35	25
Disagree	19	13
**Total**	**144**	**100**
**Confidence to use BL for teaching**		
Strongly agree	20	14
Agree	38	26
Uncertain	35	25
Strongly disagree	29	21
Disagree	20	14
No response	2	0
**Total**	**144**	**100**
**Preference for BL or traditional teaching method**		
Strongly agree	24	17
Agree	26	19
Uncertain	18	12
Strongly disagree	40	27
Disagree	35	25
No response	1	0
**Total**	144	100
**Traditional face-to-face is the best method of teaching**		
Strongly agree	27	18
Agree	30	21
Uncertain	13	9
Strongly disagree	45	32
Disagree	29	20
**Total**	**144**	**100**

*Source*: Namulondo, S., Rasweswe, M M. & Mooa, R S., 2020, *Nurse Educators’ readiness to use blended learning in public nursing education institutions in Gauteng Province, South Africa*, viewed n.d., from https://repository.up.ac.za/handle/2263/78869

BL, blended learning.

#### Psychological attitudes

Psychological readiness measured the nurse educator’s readiness and willingness to use BL, motivation to use BL, and confidence to use BL for teaching. As indicated in Table 3, 49% (*n* = 71) indicated that they are psychologically ready and willing to use BL pedagogy, 17% (*n* = 24) were still uncertain and 34% (*n* = 49) were not psychologically ready or willing to use BL. It is worth mentioning that the nurse educators in public NEIs in GP, South Africa were less motivated to use BL. The results show that 42% (*n* = 60) either strongly agreed or agreed to be motivated to use BL pedagogy during or beyond the COVID-19 pandemic, while the majority, that is, 58% (*n* = 84) were either uncertain or not motivated. Adding to concerns is that the nurse educators in general felt less confident in using BL pedagogy for teaching, as only 14% (*n* = 20) strongly agreed and 26% (*n* = 37) agreed to have confidence in using BL pedagogy. Compared to the traditional method of teaching and learning pedagogy, a larger part, that is, 52% (*n* = 75) of the nurse educators in public NEIs feel more comfortable using BL pedagogy. What is of concern is that there were 12% (*n* = 17) who were uncertain and 36% (*n* = 52) of educators who preferred the traditional face-to-face method despite the COVID-19 pandemic. Some of the nurse educators, that is, 39% (*n* = 56) felt that face-to-face teaching is the best teaching approach, while 9% (*n* = 13) were uncertain. Table 3 presents the results on psychological attitudes.

### Attitudes towards the infrastructure readiness

**FIGURE 1:**
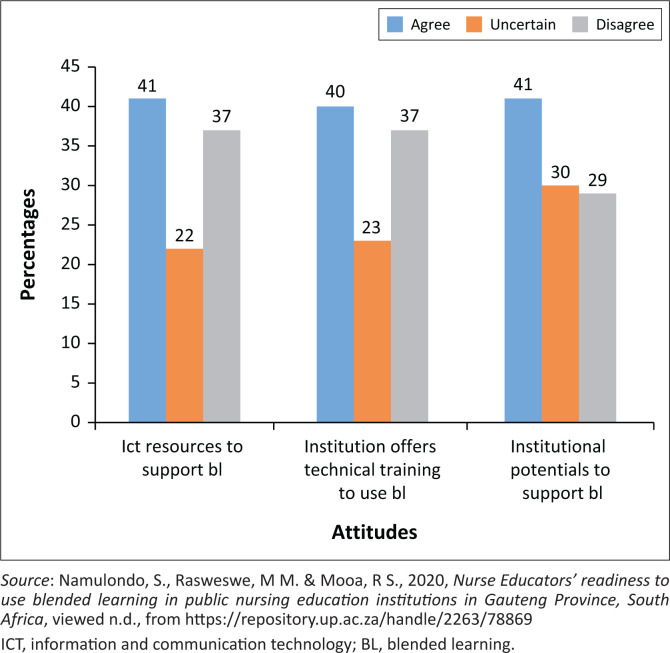
Attitudes towards the infrastructure readiness.

The questions on infrastructure dimension included availability of ICT resources to support BL pedagogy, provision of technical training to use BL by the institution, and to confirm the potential of the institution in supporting the BL once it is implemented. Data indicated that less than half of nurse educators, that is, 41% (*n* = 59) confirmed that their institution had ICT resources to facilitate BL in the classrooms, while 59% (*n* = 85) either disagreed or were uncertain about the availability of the ICT resources. Regarding the provision of technical training to staff, 40% agreed (*n* = 58), 23% (*n* = 33) were uncertain and 37% (*n* = 53) disagreed. The majority, that is, 41% (*n* = 59) indicated that their institutions do not have the potential to support BL, 30% (*n* = 43) were uncertain, while only 29% (*n* = 42) confirmed potential support. Refer to Figure 1.

### Attitudes regarding equipment readiness

**FIGURE 2:**
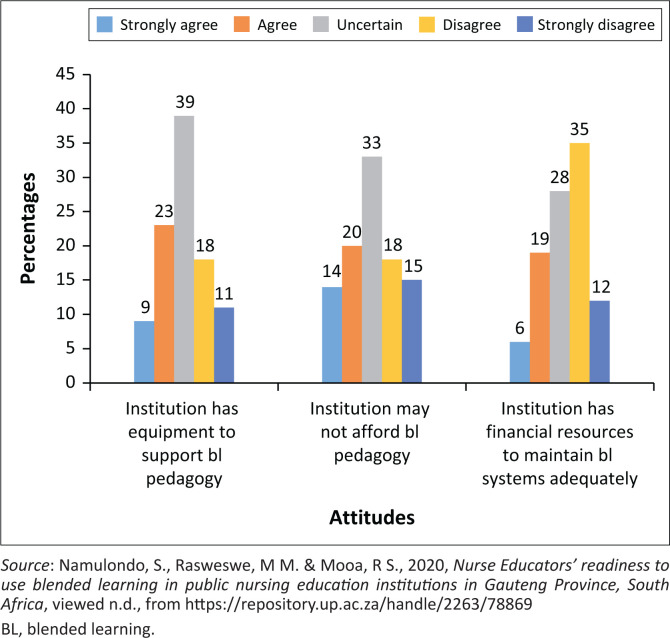
Attitudes regarding equipment’s readiness.

Attitudes regarding equipment readiness were based on the availability of basic technological devices such as laptops, computers and projectors, among other items required for the delivery of classes. It further measured attitudes regarding the availability of finances to sustain BL pedagogy. The results related to attitudes regarding equipment indicate that the nurse educators in public NEIs were not satisfied with the equipment that the NEIs are using for BL teaching and learning. As illustrated in Figure 2, 32% (*n* = 46) agreed that their institutions have effective equipment to support BL pedagogy, 39% (*n* = 56) were uncertain while 29% (42) indicated that the institutions do not have sufficient equipment to use for BL pedagogy. The respondents were also asked if the institutions have the financial ability to sustain the use of BL. The results indicated that 34% (*n* = 49) agreed that the institutions are financially stable to sustain the use of BL. Out of 144 respondents, 33% (*n* = 48) were uncertain about the financial ability, while 33% (*n* = 48) indicated that institutions do not have the financial ability to sustain the use of BL pedagogy.

## Discussion

The discussion of results with regard to the attitudes of nurse educators in the public NEIs towards the use of BL as a new norm during and beyond the COVID-19 pandemic will be described below. From the data, it was indicated that the GP public NEIs’ nurse educators are not adequately ready to use BL pedagogy in teaching and learning.

### Technological attitudes

From the data gathered, the authors could confirm that BL pedagogy was not an easy task to implement among public NEIs’ nurse educators. Although half, that is, 50% (*n* = 72) of the respondents agreed to easy use, 49% (*n* = 70) of respondents out of 144, disagreed. These results resonate with the study that found educators usually struggle to transfer study content online to use BL (Singh, Steele & Singh 2021). Singh et al. (2021) also indicated that a large number of educators and instructors experience technical challenges with the operation of online tools and accessing campus resources online. However, it is fair to consider that the selected public NEIs were still in training to implement BL pedagogy when the COVID-19 pandemic arose, which might have caused uneasiness. In agreement, Singh et al. (2021) argue that a sudden change to online teaching methods usually causes significant stress among educators, especially if they do not have experience or have minimal experience working with technology. Therefore, implementing a new teaching method in an emergency situation, as with the COVID-19 pandemic, is not a challenge as in a normal situation (Naqvi & Zehra 2021). Moreover, Jowsey et al. (2020) argue that despite sudden implementation, educators should be willing, flexible and open-minded to learn and be skilful in using BL pedagogy to support positive learning activities. If the nurse educators in our study were open-minded and willing to learn the use of BL, it would have been easy and less challenging for them to implement a BL pedagogy during and beyond the COVID-19 pandemic.

### Psychological attitudes

Psychological readiness and willingness to use BL during and beyond the COVID-19 pandemic revealed that 51% (*n* = 73) of the public NEIs’ nurse educators were either uncertain or not psychologically ready or willing to use the BL pedagogy. This might have been caused by the increased anxiety, resulted from the uncertainties and panics during COVID-19 pandemic. This might have been influenced by the fear, grief and personal loss caused by the COVID-19 pandemic (Singh & Matthees 2021). These results are supported by the study conducted in the United Arab Emirates, which revealed that it was stressful to transition to e-learning during the COVID-19 pandemic because both educators and students experienced anxiety of some sort due to the rapid changes (Mukasa et al. 2021). In India, educators reported that online teaching including BL was stressful because it is time-consuming, too formal and lacked personal touch (Nambiar 2020). Some nurse educators also indicated that they have negative attitudes towards the use of BL during the COVID-19 pandemic, which eventually affected their psychological readiness and successful uptake thereof.

The results further indicated that less than half, that is, 42% (*n* = 61) of the respondents displayed positive motivation towards the use of BL when it was introduced after the COVID-19 pandemic, while 38% (*n* = 54) and 20% (*n* = 29) of the respondents either expressed negative attitudes or were uncertain towards the use of BL pedagogy. Expression of motivation towards the use of BL enhances users’ readiness, provided the BL infrastructure and support are provided. Similarly, more than half of the participants were rarely or sometimes motivated to engage in e-learning during the COVID-19 pandemic (Mukasa et al. 2021). In addition, Khalil et al. (2020) indicated that although educators may have computer literacy skills and knowledge required to use BL pedagogy, many are still reluctant or not motivated due to their individual beliefs which may be positive or negative. The reluctance and unmotivated attitudes will therefore have a direct impact on their psychological readiness to use BL pedagogy (Ibrahim & Nat 2019).

The nurse educators’ possession of confidence held during the COVID-19 pandemic might enhance their readiness while their lack of confidence might be an indication of non-readiness to use BL pedagogy, and thus hinder the implementation beyond the COVID-19 pandemic. Coopasami et al. (2017:301) explained that nurse educators’ uptake of BL is directly influenced by their psychological temperament of confidence levels, commitment and aptitude. Contrary to our study, the findings on the impact of online learning during the COVID-19 pandemic: reported that teachers boosted the increased confidence in using technology (Nambiar 2020).

To further determine the psychological attitudes of the public NEIs’ nurse educators, they were asked to choose between BL and traditional face-to-face pedagogy. The results revealed that the respondents do not prefer BL compared to traditional face-to-face pedagogy. The results are similar to the one conducted in Malaysia, in which respondents felt that traditional face-to-face is better than BL pedagogy (Hamzah et al. 2020). Additionally, traditional face-to-face learning is associated with several benefits such as shared learning, real-time interaction between faculty–students and student–student and poses less stress compared to BL (Wadesango 2021). It is important for the nurse educators to be psychologically prepared to use BL pedagogy to deliver nursing educational content, because BL improves the students’ understanding of learning in nursing schools (Eka, Houghty & Juniarta 2019). The same authors confirmed that when BL is used among nursing students, there were positive outcomes, because they paid more interest in learning through face-to-face and online.

### Attitudes towards the infrastructure readiness

In this study, a larger number of respondents, that is, 41% (*n* = 59) reported inadequate infrastructure to enable the smooth running of BL pedagogy. Similarly, in Nigeria, poor infrastructure for technology was reported to be a challenge in implementing online teaching and learning (Apuke & Iyendo 2018). According to Jowsey et al. (2020), insufficient ICT infrastructure is one of the barriers to nurse educators’ readiness to successfully utilise BL. Furthermore, malfunctioning of ICT infrastructure may hinder uptake, especially when the nursing education institution does not have adequate ICT capacity (Rizvi et al. 2017).

The implementation of BL without adequate infrastructure in the public NEIs might have been instigated by the COVID-19 pandemic. Singh et al. (2021) confirmed that during the COVID-19 pandemic, many academic institutions transitioned to online teaching and learning without adequate infrastructure. It is, therefore, important to note that poor or a lack of online infrastructure might have created more difficulties for educators and instructors to successfully use online teaching and learning such as BL. Possession of modern computer laboratories with enough computers might improve the nurse educators’ readiness for BL pedagogy. Hence, it is suggested that academic institutions should first build appropriate infrastructure such as computer laboratories to support hybrid and BL teaching pedagogies (Singh et al. 2021).

### Attitudes regarding equipment readiness

In general, the infrastructure for technology includes necessary equipment such as computers, Wi-Fi data, software, hardware, ICT systems, among others, that are used during online teaching and learning. A prior study confirmed that ICT equipment’s accessibility can promote educators’ readiness to utilise BL to improve increased usage, provided that training is given (Bokolo et al. 2022). It was also indicated that when any NEI has adequate computers, there is a potential for successful implementation of BL pedagogy (Mukasa et al. 2021). As seen in the current study, participants in the study on teachers’ perceptions of using the BL approach for Science, Technology, Engineering and Maths (STEM)-related subjects within the fourth industrial revolution (4IR) had challenges with the implementation due to the shortage of technology-based tools within their educational milieus (Naidoo & Singh-Pillay 2020).

It indicates that inadequate or no access to appropriate equipment leads to frustrations when using BL (Mukasa et al. 2021). Inadequate equipment’s are usually due to financial difficulties, when finances are inaccessible for the NEIs to have BL systems, internet connectivity and a technical support team to assist both staff and students. According to Rizvi et al. (2017), inadequate financial sources are one of the major hurdles to using BL as it directly hinders faculty’s and nurse educators’ willingness to use BL. In support, Ibrahim and Nat (2019) argue that the non-existence of ICT tools deters the utilisation of BL among nurse educators while availability encourages its uptake.

## Limitations

The desired sample size was not achieved, and this limited generalisability to the study population. The study was also conducted only in public NEI’s in Gauteng province, South Africa, whereas larger and more samples would have been used by including private NEIs and other provinces NEI’s. Therefore, there is no generalisation of the results to other NEIs in South Africa. In addition, using a questionnaire might have limited the obtaining of rich data because the researchers could not collect any additional information through probing. Some respondents did not fully complete the questions; hence, the missing responses might have contributed differently during data analysis. Despite the limitations, this study has determined and clarified the attitudes of public NEIs’ nurse educators in GP.

## Recommendations

The concerns drawn from the results point towards the fact that the participated NEIs are still struggling with basic tools to enable the use of online platforms for teaching and learning. This suggests further research needs to be conducted with the students to provide more detailed information that will assist in determining the readiness and willingness of both nurse educators and student nurses to use BL pedagogy. It would be beneficial if NEIs can utilise the Chapnick’s readiness model to perform comprehensive readiness assessment checks to quickly recognise gaps and ensure increased uptake and sustainability of the BL pedagogy. Likewise, nurse educators’ readiness to use BL pedagogy may be promoted through the establishment of institutional technical training, psychological support, and by providing adequate infrastructure and equipment before commencement.

## Conclusion

The study aimed to determine the attitudes of nurse educators towards the use of BL as a new norm during and beyond the COVID-19 pandemic. It also revealed that although public NEIs switched to BL during the COVID-19 pandemic, they were not technically and psychologically ready. Their BL infrastructure was limited, and they also lacked some equipment to use for BL. These contributed to their lack of willingness and confidence towards the use of BL pedagogy, which ultimately impacts nurse educators’ readiness, uptake and successful implementation of BL. In general, their attitudes towards the use of BL pedagogy are negative and might impede the use beyond the COVID-19 pandemic. This study has shown the value of preparing and equipping educators with digital technology skills so that they become competent in delivering learning using online platforms. The study further highlighted the importance of establishing and supplying proper infrastructure and equipment to enable adequate digital platform learning.
